# Visualization of changes in the foveal avascular zone in both observed and treated diabetic macular edema using optical coherence tomography angiography

**DOI:** 10.1186/s40942-017-0074-y

**Published:** 2017-06-19

**Authors:** Aditya Gill, Emily D. Cole, Eduardo A. Novais, Ricardo N. Louzada, Talisa de Carlo, Jay S. Duker, Nadia K. Waheed, Caroline R. Baumal, Andre J. Witkin

**Affiliations:** 1 New England Eye Center, Tufts Medical Center, Tufts University, 800 Washington Street, Box 450, Boston, MA 02111 USA; 2 Department of Electrical Engineering and Computer Science, and Research Laboratory of Electronics, Massachusetts Institute of Technology, Cambridge, MA 02139 USA; 3 Department of Ophthalmology, Federal University of São Paulo, São Paulo, Brazil; 4 Ophthalmic Center Reference (CEROF), Federal University of Goiás, Goiânia, Brazil

**Keywords:** Diabetic macular edema (DME), Foveal avascular zone (FAZ), Optical coherence tomography (OCT), Optical coherence tomography angiography (OCTA)

## Abstract

**Background:**

Central vision loss in diabetic retinopathy is commonly related to diabetic macular edema (DME). The objective of this study was to describe changes between consecutive visits on optical coherence tomography angiography (OCTA) of the foveal avascular zone (FAZ) in eyes with DME.

**Methods:**

20 eyes from 14 patients with DME were imaged on 2 successive clinic visits separated by at least 1 month. The mean interval between visits was 3.2 months. The only intervention used was intravitreal anti-VEGF in 11 eyes; the others were observed over time without treatment. Two different readers measured FAZ area using a pseudo-automated tool in comparison to a manual tracing tool. Qualitative changes in the appearance of the vasculature surrounding the FAZ were also recorded. The retinal capillary plexus was segmented into deep and superficial plexuses, and FAZ measurements were done on the superficial, deep, and summated plexuses.

**Results:**

Pseudo-automated and manual measurements of FAZ area decreased significantly (*p* < 0.05) between visits in the deep, superficial, and summated plexuses. Qualitative analysis of vasculature surrounding the FAZ showed that most of the vascular changes (65%) over time were visible in the deep plexus, compared to 30 and 20% in the superficial and summated plexuses, respectively.

**Conclusions:**

The most significant differences in FAZ size over time were in the summated plexus (*p* < 0.001), while changes in FAZ appearance were most prominent in the deep plexus. Absolute decrease in FAZ size over visits was largest in the deep plexus. Our results demonstrate that OCTA can effectively be used to measure FAZ area in patients with DME, visualize qualitative changes in retinal vasculature, and visualize the segmentation levels at which these changes can be best appreciated. However, larger studies are needed to evaluate the reproducibility of manual and pseudo-automated measuring techniques.

## Background

Among its many health complications, diabetes is one of the leading causes of vision loss in the working age population [1]. Central vision loss in diabetic retinopathy is most commonly related to diabetic macular edema (DME); therefore DME can substantially impact quality of life [2, 3]. Alteration of the blood retinal barrier (BRB) is the hallmark of DME [4]. Factors such as ischemia, changes in blood flow, increased vascular endothelial growth factor (VEGF), oxygen-free radicals, endothelial and pericyte dysfunction, and inflammation all contribute to the breakdown of the BRB [4–6]. This disruption of the BRB in turn causes increased vascular permeability, and macular edema may present as accumulation of fluid in both the inner and outer plexiform layers of the retina, as well as the subretinal space [5].

Various imaging modalities may be utilized to help diagnose and monitor progression of DME, most notably fluorescein angiography (FA) and optical coherence tomography (OCT). FA requires intravenous dye injection to image dynamic perfusion of the retina vessels and dye leakage from increased vascular permeability [7]. OCT is able to non-invasively visualize structural features of DME including hard exudates, intraretinal or subretinal fluid accumulation, and abnormalities of the vitreomacular interface. In addition, macular thickness may be automatically and reproducibly quantified using OCT, which is useful in monitoring response to various therapies for DME [8].

OCT angiography (OCTA) is a relatively new, noninvasive tool that allows simultaneous visualization of the retinal vasculature and microstructure. It does so by mapping erythrocyte movement through the decorrelation signal between sequential OCT B-scans at the same cross section [9–11]. OCTA scans are depth-resolved and give high resolution volumetric angiograms [11]. During image acquisition of OCTA, OCT B-scan images are acquired simultaneously, therefore vascular changes on OCTA may be correlated with structural changes on corresponding OCT B-scans.

In some patients with DME, central visual loss may not only be due to the macular edema itself but also may be due to alterations in the foveal avascular zone (FAZ). Increased FAZ size in diabetics can often be visualized on FA, and is due to retinal capillary alterations in the macula [12]. Histologic features of these alterations can include, but are not limited to, formation of microaneurysms, vascular proliferation, Müller cell gliosis, preretinal damage, and damage to the outer retina [13]. However, FA is unable to visualize changes in a depth-resolved fashion in the superficial and deep capillary plexuses, and the FAZ can be difficult to visualize in patients with DME due to dye leakage and obscuration of macular vascular details [14]. In addition, it is unclear what relationship the appearance and disappearance of intraretinal fluid has on the anatomy of the FAZ in eyes with DME, as it is difficult to perform fluorescein angiography studies on patients at consecutive visits due to its invasive and time consuming nature. These issues are in large part circumvented when using OCTA technology.

The purpose of this study is to utilize OCTA to analyze the appearance and size of the FAZ in eyes with DME over time and to describe whether these changes can be seen in a depth-resolved fashion.

## Methods

This was a retrospective, observational case series completed under the tenets of the Declaration of Helsinki and the Health Insurance Portability and Accountability Act of 1996. The Institutional Review Board of Tufts Medical Center gave approval for the study, and subjects were recruited from the retina service of New England Eye Center at Tufts Medical Center in Boston, MA between August 2014 and March 2015. All subjects provided written consent before OCTA imaging using a commercially available spectral domain OCT (SD-OCT) device (RTVue XR with prototype AngioVue software, Optovue Inc., Fremont, CA). This SD-OCT operates at 70,000 A-scans per second to acquire OCTA volumes consisting of 304 × 304 A-scans over an area of 3 × 3 mm. Corresponding structural OCT B-scans are obtained concurrently.

All patients had DME diagnosed with fundus examination by a retina specialist and confirmed with standard 6-mm SD-OCT imaging. All patients also completed OCTA imaging at two or more visits spaced at least 1 month apart. Subjects with concomitant, confounding retinal diagnoses, such as age-related macular degeneration, retinal vascular occlusion, and central serous chorioretinopathy, were excluded. OCTA scans were considered to be of poor quality if the FAZ could not be delineated, and poor quality images were excluded.

**Fig. 1:**
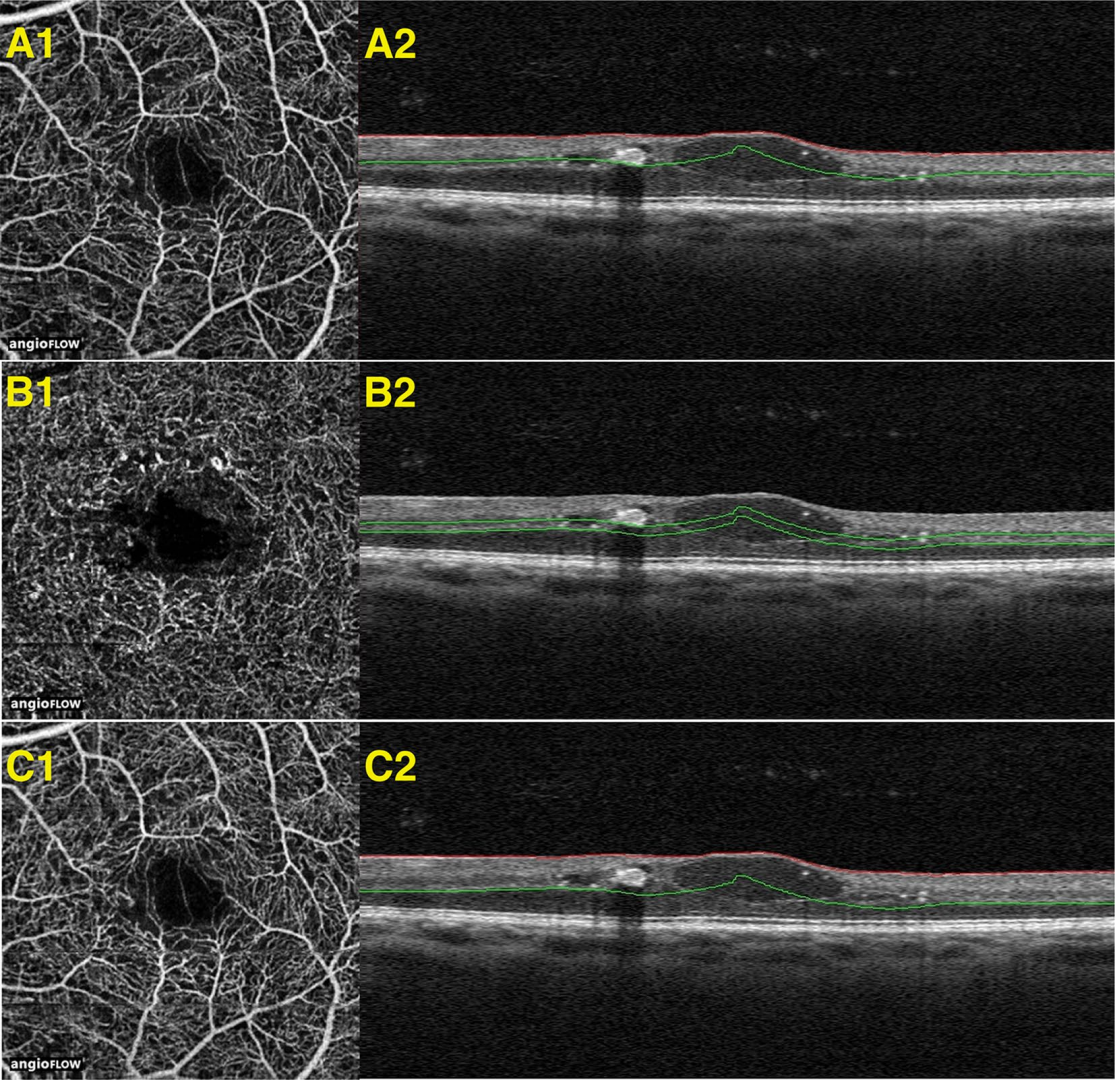
Segmentation of superficial, deep, and summated retinal vascular plexus. OCT-B scans with segmentation shown in correspondence with OCTA images delineating the area of the vasculature being examined. These images are from a patient diagnosed with diabetic macular edema. **A1**, **A2** demonstrate OCTA image with its corresponding OCT B-scan, respectively, at the superficial plexus. **B1**, **B2** demonstrate OCTA image with its corresponding OCT B-scan, respectively, at the deep plexus. **C1**, **C2** demonstrate OCTA image with its corresponding OCT B-scan, respectively, at the summated plexus

The FAZ was defined as the area encompassing the central fovea, where there were no clear and demarcated vessels seen on OCTA. The FAZ was evaluated using 3 × 3-mm OCT angiograms from each study eye. OCTA images were segmented at the level of the superficial, deep, and summated plexus of the inner retinal vasculature. Automated segmentation algorithms on the AngioVue software were initially used to help generate segmentation lines between the superficial and deep retinal capillary plexuses. However, since altered retinal contour due to DME can cause segmentation error, the automatic segmentation lines were then manually moved anteriorly or posteriorly to fit the following criteria: *the superficial plexus* was calculated between the internal limiting membrane and a line 15 μm deep to the outer border of the inner plexiform layer*, the deep plexus* was calculated between the line offset 15 μm deep to the inner plexiform layer and a line offset 70 μm deep to the outer boundary of the outer plexiform layer, and *the summated plexus* was calculated between the internal limiting membrane and a line offset 70 μm deep to the outer boundary of the outer plexiform layer (Fig. 1) [15]. It is worth noting that we used one machine for our scans and to follow up with our patients, as there may be inconsistencies between data collected among multiple OCTA machines [16].

The 3 × 3-mm angiograms were evaluated by two trained readers (AG, EC) at two consecutive patient visits. The extent of the edema was assessed visually by examining OCT angiograms in conjunction with co-registered OCT-B scans, *en*-*face* structural OCT, and retinal thickness maps, if available. For each visit, two readers evaluated the size of the FAZ by manually delineating the border using the shape tracing tool on Image J software (National Institutes of Health, Bethesda, Maryland, USA; available at http://rsb.info.nih.gov/ij/index.html). The FAZ was also evaluated using a pseudo-automated tool built into the AngioVue software. By clicking at the center of the FAZ, this algorithm automatically selected a focal area of non-flow activity corresponding to the FAZ, with automatic measurement of the non-flow area. Both readers used this pseudo-automated method by clicking at the center of the FAZ as many times as deemed necessary for each scan until the reader felt that the software best highlighted and most closely represented the true area of the FAZ. The process to measure FAZ area using the pseudo-automated method in the superficial and deep plexus, as well as manually delineate the superficial plexus and measure its associated FAZ area took roughly 5 min per image. The length of time to perform this analysis may increase if the reader is not familiar with the interpretation of OCTA images or the software associated with the device.

Two readers also recorded descriptive changes (AG, AW) seen in retinal vasculature between visits on OCTA images of the superficial, deep, and summated plexuses. This was best done by directly overlaying the OCTA image from the second visit over the image from the first visit, and slowly increasing/decreasing the transparency of it to help appreciate microscopic changes in the retinal vasculature. At visits with greater amounts of macular edema, vessels were described as being either displaced by fluid-filled spaces or disappearing between consecutive visits.

### Statistical analysis

Statistical analysis was performed with Stata 13 software (StataCorp. 2013. *Stata Statistical Software: Release 13*. College Station, TX: StataCorp LP). Intraclass correlation coefficient (ICC) was calculated between both readers for both automated and manual measurements of the FAZ. ICC between readers showed good reliability (between 0.64 and 0.99). A Wilcoxon Signed-Rank test was performed to compare FAZ measurements between the two visits. *p* values of <0.05 were considered statistically significant.

## Results

A total of 22 eyes from 16 patients who had DME were imaged on two consecutive visits between August 2014 and January 2017. Two eyes from 2 patients were excluded in the study because of residual motion artifact that caused the images to be of poor quality such that the FAZ could not be clearly delineated. The remaining twenty eyes from 14 patients were used for analysis. Seven women and seven men were included in the study with a mean age of 61 years (range 34–72 years, SD ±10 years). Eleven patients were Caucasian and three patients were African-American. Six eyes had DME associated with proliferative diabetic retinopathy and fourteen eyes had DME associated with non-proliferative diabetic retinopathy. Mean time interval between the two visits used in the study was 3.2 months (range 1–9 months). Between the two visits, nine of the eyes received no treatment, six eyes received one injection of intravitreal aflibercept, two eyes received two injections of intravitreal bevacizumab, and three eyes received two injections of intravitreal ranibizumab. No eyes were treated with laser photocoagulation or other treatment modalities between visits.

**Fig. 2:**
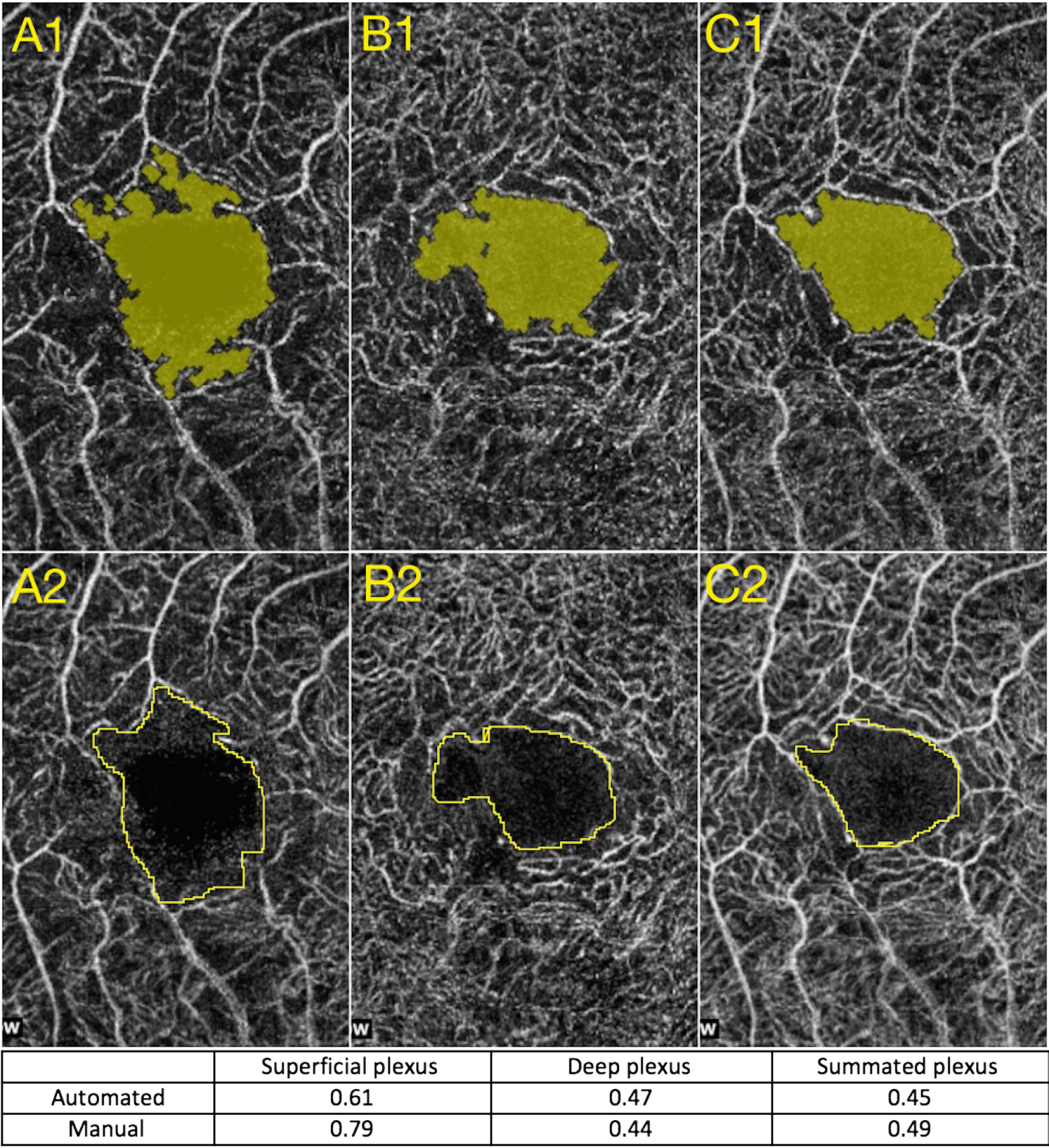
Comparison of partially automated versus manual measurements of the FAZ. **A1**, **B1**, and **C1** represent automated measurements taken at the superficial, deep, and summated plexuses, respectively. **A2**, **B2**, and **C2** represent manual drawings of the measurements taken at the superficial, deep, and summated plexuses respectively

**Fig. 3:**
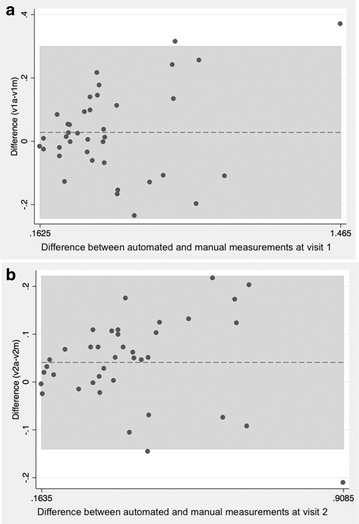
Bland-Altman plot comparing the partially automated and manual measurement technique for visit 1 (**a**) and visit 2 (**b**). The x-axis signifies the average in FAZ area (mm^2^) while the y-axis signifies the difference between the two measurement techniques

**Fig. 4:**
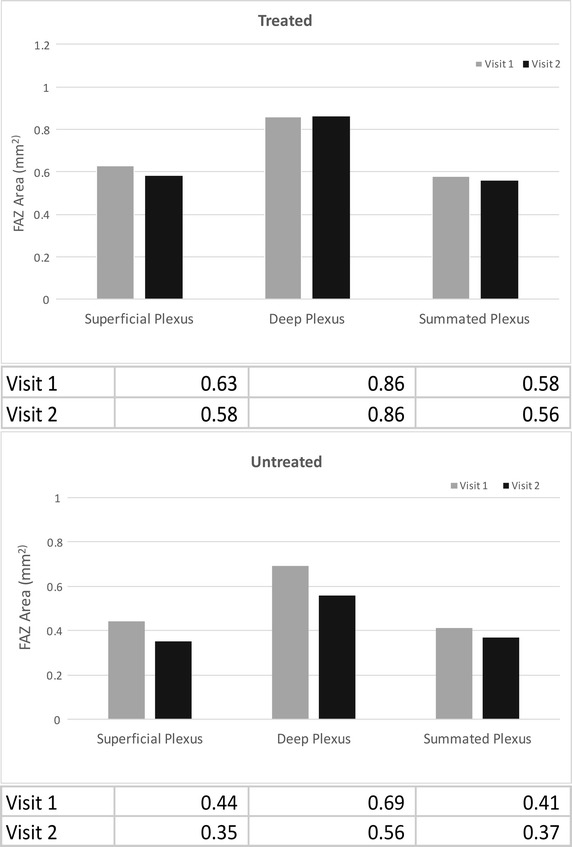
Average FAZ size in patients treated with anti-VEGF and untreated patients. The average values are listed in the table below each* bar graph*. The average FAZ value in treated patients was larger than the average FAZ value in untreated patients in superficial, deep, and summated plexuses

 Figure 2 shows comparisons between pseudo-automated and manual FAZ measurements, as well as the calculated areas of FAZ size. Intra-class coefficients were calculated between the two readers for both visits, for both methods of FAZ measurements, and for all three superficial, deep, and summated plexus measurements. After establishing strong ICC values between the readers (Range 0.90–0.99 for pseudo-automated measurements, Range 0.64–0.89 for manual measurements), reader one’s measurements were used to calculate ICC (Range 0.92–0.97) for correlation between partially automated and manual FAZ measurements. Bland–Altman plots were constructed in order to compare the two FAZ area measurement techniques for the two visits (Fig. 3). The variation between the two methods increased as the size of the average FAZ area increased. Finally, the difference in FAZ area between the two visits was calculated for all three plexuses using the pseudo-automated and manual FAZ measurement techniques (Table 1). There was a statistically significant reduction (*p* < 0.05) in the size of the FAZ between the two visits at all segmentation levels for both manual and pseudo-automated measurements. The most statistically significant difference in FAZ area between visits was measured in the summated plexus using both the pseudo-automated (*p* = 0.0006) and manual (*p* = 0.0008) methods. The greatest absolute difference in FAZ area between visits was seen in the deep plexus using both the manual method (decrease of 0.09 mm^2^, *p* = 0.019) and the pseudo-automated method (decrease of 0.07 mm^2^, *p* = 0.029). Figure 4 shows the average FAZ size for both visits in the treated (with anti-VEGF injection) and untreated groups. Average FAZ size was larger in the treated group than in the untreated group across all categories. Further statistical analysis of the treated and untreated data was not conducted because of the small sample size.

**Table 1:** Change in FAZ area between visits for all patients

	Automated FAZ measurement	Manual FAZ measurement
Average decrease in FAZ area (mm^2^)—deep plexus	0.09 (*p* = 0.019)	0.071 (*p* = 0.029)
Average decrease in FAZ area (mm^2^)—superficial plexus	0.057 (*p* = 0.031)	0.062 (*p* = 0.016)
Average decrease in FAZ area (mm^2^)—summated plexus	0.037 (*p* = 0.0006)	0.035 (*p* = 0.0008)

The mean change in the FAZ area (in mm^2^) between the two visits at the deep, superficial, and summated plexuses. *p* values are shown in parenthesis and were calculated using paired *t* tests

**Fig. 5:**
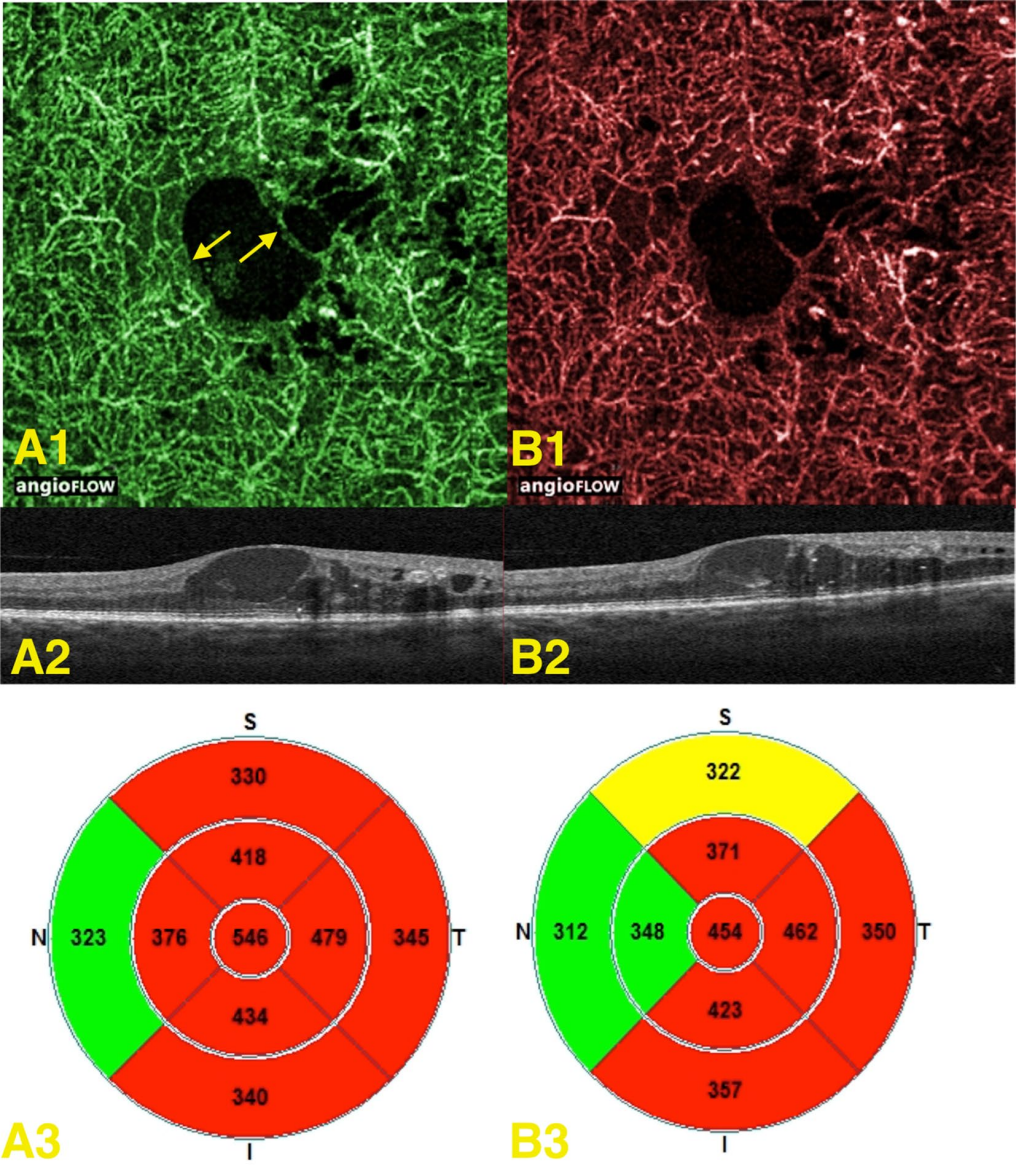
Displacement of vessels due to fluid filled spaces in the deep plexus. **A1** expresses the OCT-angiogram of the initial patient visit with arrows indicating the displacement; **A2**, **A3** represent the corresponding OCT B-scan and retinal thickness map, respectively. **B1** expresses the OCT-angiogram of the second patient visit where vessels appear to be more central in the FAZ; **B2**, **B3** represent the corresponding OCT B-scan and retinal thickness map, respectively

**Fig. 6:**
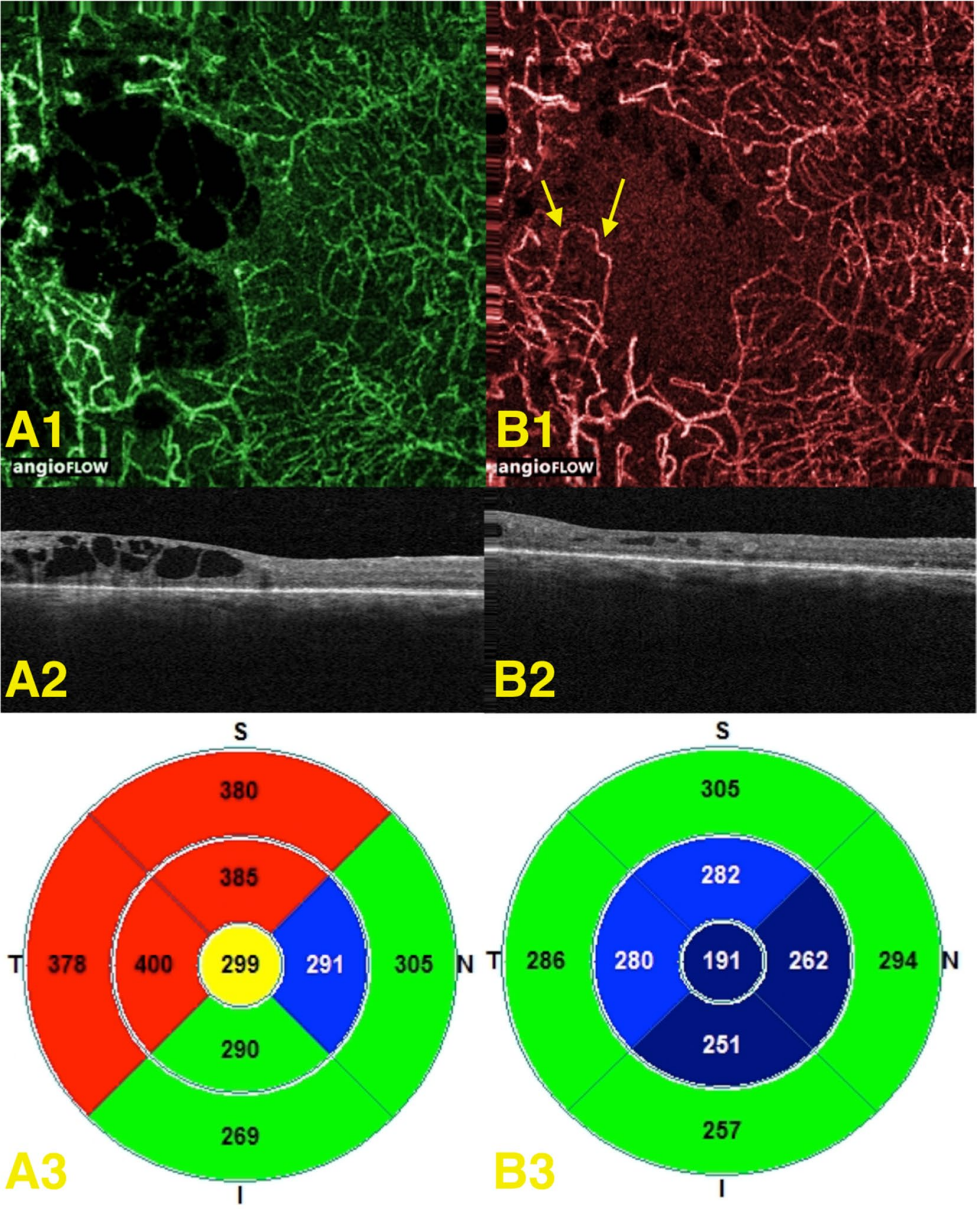
Disappearance and reappearance of flow signal in the deep plexus. **A1** expresses the OCT angiogram of the initial patient visit; **A2**, **A3** represent the corresponding OCT-B scan and retinal thickness map, respectively. **B1** expresses the OCT-angiogram of the second patient visit, with *arrows* pointing to vessels that were not found in the in the initial visit due to loss of flow signal. **B2**, **B3** represent the corresponding OCT-B scan and retinal thickness map, respectively

Qualitative measurements were also performed. A decrease in FAZ size between visits could be described in one of two ways: some eyes had a decrease in FAZ size between visits due to a decrease in vessel displacement coinciding with disappearance of intraretinal fluid (Fig. 5), while other eyes had a decrease in FAZ size between visits due to a reappearance of flow signal coinciding with disappearance of intraretinal fluid (Fig. 6). These qualitative changes were visible between visits on OCTA images of the deep plexus in 13/20 (65%) eyes. However, qualitative changes between visits were only visible in 6/20 (30%) OCTA images of the superficial plexus and 4/20 (20%) OCTA images of the summated plexus.

## Discussion

OCTA is a noninvasive imaging modality that may help augment characterization of DME and its potential effects on retinal microvasculature, particularly on the FAZ. In the current study, we compared manual and pseudo-automated measurements of the FAZ in the deep, superficial, and summated retinal capillary plexuses, and compared these measurements between two consecutive visits. The most statistically significant difference in the FAZ area between visits was measured using OCTA images of the summated capillary plexus. We also examined the effects of the intraretinal fluid on the retinal capillaries surrounding the FAZ, and found that the most apparent effects of edema on these vessels were observed in the deep retinal plexus.

Fluorescein angiography (FA) has previously demonstrated that FAZ area is increased in patients with DME compared to normal healthy controls [12, 17, 18]. However, FA has several limitations including its invasive nature, inability to image the deep retinal capillary plexus [10, 19], and needing for dye to clear before back-to-back FA’s can be performed. OCTA is non-invasive, can image retinal vasculature and microanatomy simultaneously, and is able to visualize the entire retinal capillary network in a depth-resolved manner. OCTA may therefore provide supplementary anatomical information that helps to better understand the pathology and progression of DME, and the relationship between macular edema and the perifoveal vasculature.

Our data is consistent with and complements some recently published papers that also utilized OCTA to study FAZ area in diabetic patients [20–23]. These papers compared OCTA of patients with diabetic retinopathy (DR) with healthy controls and found FAZ area to be significantly larger in the DR group. Additionally, Di et al. [20] found that the FAZ area was significantly larger in the clinically significant macular edema (CSME) group versus the non-CSME group in a singular plexus, while Freiberg et al. [21] and Takase et al. [22] examined both the superficial and deep plexuses and found more pronounced changes in FAZ area in the deep plexus compared to healthy eyes. Furthermore, the latter study also found a statistically significant enlargement in FAZ area in diabetic eyes, regardless of the presence of retinopathy [22]. This suggests that changes within the FAZ may occur at early stages of diabetic retinopathy.

However, these studies were limited in the fact that they only observed the FAZ areas in the superficial and/or deep plexuses using a singular measurement method. Furthermore, some of these studies did not evaluate the change in FAZ size over time in patients with diabetic macular edema. In our study, we expanded on their findings and examined all three plexuses (deep, superficial, and summated) in the same set of patients with DME over two consecutive visits. Along with strong reproducibility of FAZ measurements between two readers using manual and partially automated methods, we also found a significant difference in FAZ size at all segmentation levels between two consecutive visits using a pseudo-automated and manual measurements, with the highest significance in the summated plexus. In addition, the difference between manual and partially automated measurements increased in eyes with larger FAZ areas (Fig. 3), suggesting that correlation between the two techniques may diminish between methods when measuring eyes with larger FAZ areas. Thus, the manual method may be preferred in this situation due to better control in outlining the desired area(s).

The most statistically significant difference between the two visits was seen when using FAZ measurements of the summated plexus. It is possible that the FAZ may be more reproducibly measured using the summated retinal capillary plexus in patients with DME, as the differentiation and segmentation of the superficial and deep plexuses may be more difficult when the retinal anatomy is distorted in diseases such as DME. Additionally, manual delineation of the border between superficial and deep retinal capillary plexuses may be less prone to segmentation error than the automatic segmentation of the superficial and deep plexuses provided via software integral to the OCTA device.

Secondly, we found that the largest decrease in FAZ area between visits was seen in the deep plexus using both manual and pseudo-automated methods of FAZ measurement. As discussed above, previous studies have found the FAZ to be larger in the deep plexus compared to the superficial plexus in eyes with DME, suggesting that macular edema may preferentially effect the deep capillary plexus [24]. Thus, clearing of macular edema may explain why there was a greater decrease in FAZ area over consecutive visits in the deep plexus. Furthermore, we speculate that OCTA is able to noninvasively visualize the effect of the anti-VEGF injections on the retinal microvasculature [25]. Figure 4 shows that the treated group had a larger average FAZ size at all segmentation levels than the untreated group. However, we cannot conclude this for certain due to the small number of patients treated with anti-VEGF in our sample size.

We also found that qualitative changes in the retinal vasculature over time, evident as either displacement of retinal capillaries or disappearance of retinal capillaries due to increase in DME, were most apparent in OCTA images of the deep plexus; these types of changes were apparent in the deep capillary plexus in 65% (13/20) eyes. These changes may have been due to remodeling of the vessels, vessel displacement, capillary closure (mechanisms for both transient and permanent vasculature obstruction have been suggested in diabetic retinas) [26], a decrease in flow velocity that may have fallen below the threshold level distinguishing low flow from noise on OCTA, or a combination of these factors. Conversely, these changes may have been due to difficulty in segmentation of the deep from the superficial plexus as the amount of retinal edema varied over time, or due to artifact innate to the OCTA technique.

There were several limitations to this study. Foremost, the small sample size, retrospective nature, and lack of a healthy control group of the study make it difficult to generalize our findings. We were also limited in the number of cases with images of acceptable quality for accurately measuring the FAZ; several cases were excluded due to poor image quality. Future studies are warranted to evaluate the ability of OCTA to acquire high quality images in patients with DME. Images of lower quality may be found in patients with poor fixation and visual acuity, however, the primary aim of this study was not to correlate FAZ size with visual acuity. Previous studies have found either a positive correlation or no correlation between the degree of FAZ enlargement in diabetics and the loss of visual acuity [22, 27, 28]. Larger and better-controlled studies with longer follow-up are needed to help confirm and expand on these findings. Secondly, we were unable to compare FA and OCTA images in our study because of the retrospective nature of the study. Few of the patients included had same-day FA and OCTA studies, and none of the patients in our sample had FA studies performed at consecutive visits. Thirdly, high quality OCTA imaging requires patients to remain stable for several seconds during scanning in order to obtain high quality images, which may be difficult for patients with poor fixation. Finally, automated segmentation software may fail with patients in edema when examining each plexus individually. Software that would allow for careful manual adjustment of segmentation lines to adjust for changes in retinal morphology may be most useful in patients with macular edema. The enlarged FAZ in the deep plexus could be due to (a) vascular changes secondary to DME occur more prominently in the deep plexus or (b) fluid in DME may cause shadowing on OCTA and loss of OCT signal at deeper segmentations. This may cause the FAZ to appear larger in the deep plexus, but it is possible that the apparent enlargement in the FAZ could also be due to artifact. Our results suggest that the summated plexus may be best segmentation for visualizing changes in the FAZ while minimizing the effect of shadowing artifact.

## Conclusion

OCTA is a can be used to visualize the retinal capillary network in three dimensions while simultaneously generating corresponding structural *en face* and cross-sectional OCT B-scans. In this study, we demonstrate that OCTA can measure FAZ area in patients with DME but larger studies are warranted to evaluate the reproducibility of the manual and pseudoautomated measuring techniques. Additionally, we demonstrate that differences in FAZ size at consecutive visits using OCTA were most significant when viewing images of the summated retinal capillary plexus, while qualitative changes in the appearance of the vasculature surrounding the FAZ may be best visualized in the deep plexus. We also found the largest change in FAZ area over time occurred in the deep plexus, which is consistent with current literature. We believe that it is important to understand and visualize the progression of DME [29], as OCTA may eventually play a role in therapeutic management of DME.]

## Abbreviations

DME: diabetic macular edema
OCTA: optical coherence tomography angiography
FAZ: foveal avascular zone
BRB: blood retinal barrier
VEGF: vascular endothelial growth factor
OCT: optical coherence tomography
FA: fluorescein angiography
DR: diabetic retinopathy
ICC: intraclass correlation coefficient
CSME: clinically significant macular edema

## References

[1] Varma R, Bressler NM, Doan QV, Gleeson M, Danese M, Bower JK (2014). Prevalence of and risk factors for diabetic macular edema in the United States. JAMA Ophthalmol..

[2] Hariprasad SM, Mieler WF, Grassi M, Green JL, Jager RD, Miller L (2008). Vision-related quality of life in patients with diabetic macular oedema. Br J Ophthalmol.

[3] Kempen JH, O’Colmain BJ, Leske MC, Haffner SM, Klein R, Moss SE (2004). The prevalence of diabetic retinopathy among adults in the United States. Arch Ophthalmol.

[4] Das A, McGuire PG, Rangasamy S (2015). Diabetic macular edema: pathophysiology and novel therapeutic targets. Ophthalmology.

[5] Zhang X, Zeng H, Bao S, Wang N, Gillies MC (2014). Diabetic macular edema: new concepts in patho-physiology and treatment. Cell Biosci..

[6] Bhagat N, Grigorian RA, Tutela A, Zarbin MA (2009). Diabetic macular edema: pathogenesis and treatment. Surv Ophthalmol.

[7] Rabbani H, Allingham MJ, Mettu PS, Cousins SW, Farsiu S (2015). Fully automatic segmentation of fluorescein leakage in subjects with diabetic macular edema. Investig Ophthalmol Vis Sci..

[8] Salz DA, Witkin AJ (2015). Imaging in diabetic retinopathy. Middle East Afr J Ophthalmol..

[9] de Carlo TE, Bonini Filho MA, Chin AT, Adhi M, Ferrara D, Baumal CR (2015). Spectral-domain optical coherence tomography angiography of choroidal neovascularization. Ophthalmology.

[10] de Carlo TE, Salz DA, Waheed NK, Baumal CR, Duker JS, Witkin AJ (2015). Visualization of the retinal vasculature using wide-field montage optical coherence tomography angiography. Ophthalmic Surg Lasers Imaging Retina.

[11] Matsunaga D, Yi J, Puliafito CA, Kashani AH (2014). OCT angiography in healthy human subjects. Ophthalmic Surg Lasers Imaging Retina.

[12] Conrath J, Giorgi R, Raccah D, Ridings B (2005). Foveal avascular zone in diabetic retinopathy: quantitative vs qualitative assessment. Eye.

[13] Weerasekera LY, Balmer LA, Ram R, Morahan G (2015). Characterization of retinal vascular and neural damage in a novel model of diabetic retinopathy. Investig Ophthalmol Vis Sci..

[14] Weinhaus RS, Burke JM, Delori FC, Snodderly DM (1995). Comparison of fluorescein angiography with microvascular anatomy of macaque retinas. Exp Eye Res.

[15] Yu S, Lu J, Cao D, Liu R, Liu B, Li T (2016). The role of optical coherence tomography angiography in fundus vascular abnormalities. BMC Ophthalmol..

[16] Magrath GN, Say EA, Sioufi K, Ferenczy S, Samara WA, Shields CL (2016). Variability in foveal avascular zone and capillary density using optical coherence tomography angiography machines in healthy eyes. Retina.

[17] Samara WA, Say EA, Khoo CT, Higgins TP, Magrath G, Ferenczy S (2015). Correlation of foveal avascular zone size with foveal morphology in normal eyes using optical coherence tomography angiography. Retina.

[18] Carpineto P, Mastropasqua R, Marchini G, Toto L, Di Nicola M, Di Antonio L (2016). Reproducibility and repeatability of foveal avascular zone measurements in healthy subjects by optical coherence tomography angiography. Br J Ophthalmol.

[19] Mendis KR, Balaratnasingam C, Yu P, Barry CJ, McAllister IL, Cringle SJ (2010). Correlation of histologic and clinical images to determine the diagnostic value of fluorescein angiography for studying retinal capillary detail. Investig Ophthalmol Vis Sci..

[20] Di G, Weihong Y, Xiao Z, Zhikun Y, Xuan Z, Yi Q (2015). A morphological study of the foveal avascular zone in patients with diabetes mellitus using optical coherence tomography angiography. Graefe’s Arch Clin Exp Ophthalmol..

[21] Freiberg FJ, Pfau M, Wons J, Wirth MA, Becker MD, Michels S (2015). Optical coherence tomography angiography of the foveal avascular zone in diabetic retinopathy. Graefe’s Arch Clin Exp Ophthalmol..

[22] Takase N, Nozaki M, Kato A, Ozeki H, Yoshida M, Ogura Y (2015). Enlargement of foveal avascular zone in diabetic eyes evaluated by en face optical coherence tomography angiography. Retina.

[23] Al-Sheikh M, Akil H, Pfau M, Sadda SR (2016). Swept-source OCT angiography imaging of the foveal avascular zone and macular capillary network density in diabetic retinopathy. Invest Ophthalmol Vis Sci.

[24] Coscas F, Sellam A, Glacet-Bernard A, Jung C, Goudot M, Miere A (2016). Normative data for vascular density in superficial and deep capillary plexuses of healthy adults assessed by optical coherence tomography angiography. Investig Ophthalmol Vis Sci..

[25] Fogli S, Mogavero S, Egan CG, Del Re M, Danesi R (2015). Pathophysiology and pharmacological targets of VEGF in diabetic macular edema. Pharmacol Res.

[26] Miwa Y, Murakami T, Suzuma K, Uji A, Yoshitake S, Fujimoto M (2016). Relationship between functional and structural changes in diabetic vessels in optical coherence tomography angiography. Sci Rep..

[27] Tam J, Dhamdhere KP, Tiruveedhula P, Lujan BJ, Johnson RN, Bearse MA (2012). Subclinical capillary changes in non-proliferative diabetic retinopathy. Optom Vis Sci.

[28] Nelson DA, Burgansky-Eliash Z, Barash H, Loewenstein A, Barak A, Bartov E (2011). High-resolution wide-field imaging of perfused capillaries without the use of contrast agent. Clin Ophthalmol..

[29] Matsunaga DR, Yi JJ, De Koo LO, Ameri H, Puliafito CA, Kashani AH (2015). Optical coherence tomography angiography of diabetic retinopathy in human subjects. Ophthalmic Surg Lasers Imaging Retina.

